# Minimally Invasive Electrochemical Patch-Based Sensor System for Monitoring Glucose and Lactate in the Human Body—A Survey-Based Analysis of the End-User’s Perspective

**DOI:** 10.3390/s20205761

**Published:** 2020-10-11

**Authors:** Roman Holzer, Wilhelm Bloch, Christian Brinkmann

**Affiliations:** 1 Institute of Cardiovascular Research and Sport Medicine, German Sport University Cologne, 50933 Cologne, Germany; roman.holzer@protonmail.com (R.H.); w.bloch@dshs-koeln.de (W.B.); 2 IST University of Applied Sciences, 40233 Düsseldorf, Germany

**Keywords:** minimally invasive biosensor, wearable sensor, microneedle, biomarker, electrochemical sensor, real-time measurement, dermal interstitial fluid

## Abstract

*Background:* Wearable electrochemical sensors that detect human biomarkers allow a comprehensive analysis of a person’s health condition. The “electronic smart patch system for wireless monitoring of molecular biomarkers for health care and well-being” (ELSAH) project aims to develop a minimally invasive sensor system that is capable of continuously monitoring glucose and lactate in the dermal interstitial fluid in real time. It is the objective of the present study to compare the intended ELSAH-patch specifications with the expectations and requirements of potential end-users at an early stage during the development phase. *Methods:* A questionnaire addressing different aspects of the ELSAH-patch was filled out by 383 respondents. *Results:* The participants stated a high general demand for such a system, and they would use the ELSAH-patch in different health care and physical fitness applications. The preferred terminal device for communication with the sensor would be the smartphone. An operating time of 24 hours would be sufficient for 55.8% of the users (95%-CI: 50.3–61.3%), while 43.5% of them (95%-CI: 38.0–48.9%) would prefer a lifetime of several days or more. The software should have a warning function, especially for critical health conditions. Since the measured personal data would be highly sensitive, the participants called for high standards for data security and privacy. *Conclusion:* In general, the participants’ responses on their expectations and requirements were well in line with the intended specifications of the ELSAH-patch system. However, certain technical aspects such as the lifetime, data security and accuracy require special attention during its development.

## 1. Introduction

In recent years, the development and application of wearables to monitor health and well-being has increased significantly in scientific as well as commercial settings. Driven by the growing functionality and affordability of miniaturized electronics, the widespread proliferation of smartphones and the growing consumer awareness of health and wellness, these wearables have rapidly become a prime example of electronic intelligent systems (ESS) with high market potential [1]. Already available wearable sensing technologies rely on mechanical, electrical or optical methods to measure certain parameters such as acceleration, heart rate, heart rate variability, blood oxygen saturation, temperature, etc. [2].

For a comprehensive analysis of health, performance or stress levels, the measurement of physical parameters and vital signs is not sufficient. The continuous real-time detection of relevant human biomarkers using wearable chemical sensors enables more in-depth monitoring and medical diagnosis for the user [3]. A biomarker can be defined as “a characteristic that is objectively measured and evaluated as an indicator of normal biological processes, pathogenic processes, or pharmacologic responses to a therapeutic intervention” [4].

Although blood is generally considered the gold standard biofluid for diagnostics and analyses of biomarkers, the development of innovative wearable technologies has focused on non-invasive approaches because blood sampling is incompatible in personal (non-medical) health care settings. Sweat, saliva or ocular fluid have been identified as alternative body fluids for biomarker analyses [5,6,7,8].

However, systems associated with the non-invasive sampling type face specific challenges [1,9]. Contamination (sweat and saliva), volatility/evaporation (sweat), large fluctuations in pH (saliva), emotion-related changes in composition (ocular fluid) and very low concentrations of biomarkers (sweat and ocular fluid) are just a few of the factors hindering progress in the development of wearable chemical sensor systems [3,10].

One possible way out of these problems is to opt for minimally invasive measurement in the dermal interstitial fluid (ISF) instead. The dermal ISF can be accessed pain-free using minimally invasive microneedles [11] and is not susceptible to contamination [12]. Furthermore, the dermal ISF has been proven to be pH stable, and its biomarker concentrations are highly similar to the reference levels in blood [13]. Consequently, minimally invasive measuring in the dermal ISF using microneedle-based sensors is the most promising approach for integrating the continuous monitoring of biomarkers into wearables for health care and well-being.

The EU-funded “electronic smart patch system for wireless monitoring of molecular biomarkers for health care and well-being” (ELSAH) project [14], which is coordinated by the AIT, Austrian Institute of Technology, with a project consortium of interdisciplinary partners, aims to develop a flexible and integrated smart patch-based wearable sensor system (“ELSAH-patch”). The size of the ELSAH-patch should be as small as possible and should not exceed the following dimensions: 4 cm × 4 cm × 4 mm (L × W × H). The ELSAH-patch is designed to electrochemically quantify several molecular biomarkers simultaneously by using a minimally invasive microneedle-based sampling method. By integrating the microneedle biosensor with a microchip, a printed battery and printed electronics (interconnects and antenna structures), the ELSAH-patch will be able to operate completely independently. Data of the autonomous measurements will be transmitted safely and wirelessly to the user’s mobile phone. In a first step and to demonstrate the technical feasibility of the ELSAH-patch, the two biomarkers glucose and lactate were selected, as they are two of the most established and prominent biomarkers for supporting a healthy lifestyle [15,16].

Several devices are already on the market for the minimally invasive monitoring of glucose, for example, the FreeStyle Libre 2 system from Abbott Laboratories Ltd. or the Dexcom G6 system from Dexcom Inc. These devices only measure glucose (in the subcutaneous ISF), and applying the sensor might be painful. By contrast, the ELSAH-patch should provide a platform for multiplexed measurements of several biomarkers in real time, and its use should be pain-free.

Since developers and possible end-users of the ELSAH-patch system may have different perspectives on the required specifications and demands, it is important to incorporate the target group’s expectations and requirements into the development process at an early stage to optimize the product for the market [17]. For this reason, an end-user survey was conducted. 

## 2. Materials and Methods

The survey targeted potential end-users of the ELSAH-patch and intended to identify the expectations on and requirements for such a system. For this purpose, a questionnaire was developed which covered different aspects of the handling and use of the ELSAH-patch. The questionnaire was used as an online version on "surveymonkey.com". The paper-and-pencil method was used as an alternative. Data were collected over a period of 3 months from 06/2019 until 09/2019. To be included in the final data set, at least 80% of the questionnaire had to be completed.

### 2.1. Subjects

The participants had to be 18 years or older. No further exclusion criteria were defined, in order to achieve the highest possible number of responses. All subjects participated voluntarily without monetary incentive and provided written consent for inclusion in the study.

### 2.2. Questionnaire

The questionnaire consisted of 57 questions and was divided into six separate sections: (1) anthropometric/sociodemographic data and experience with glucose and lactate measurements, (2) an explanation of the ELSAH project, (3) user behavior and acceptance, (4) technical aspects and design, (5) software and data security and (6) pricing (see Supplemental Data File). The participants were informed about the purpose of the study, the estimated time it would take to complete the questionnaire, and the declarations on confidentiality, data privacy and the researchers’ contact details. All questions were implemented as 5-point Likert rating scales, single/multiple choice (with the option to add unlisted answers) or open-ended questions [18].

### 2.3. Validation of the Questionnaire

Prior to the survey, the questionnaire was reviewed by three scientific colleagues from different disciplines to ensure that the questions were understandable and unambiguous. Once their feedback had been incorporated, the questionnaire was distributed to individuals from the potential target groups to identify possible problems. This preliminary group included sports scientists, coaches and patients with diabetes mellitus (who often use continuous glucose-measuring systems), but also persons who were unfamiliar with sports and exercise and/or self-monitoring health variables to eliminate comprehension problems in relation to technical terms or expressions. The group’s feedback was used to adapt the questionnaire into its final version. The average time required to complete the questionnaire was 10–12 minutes.

### 2.4. Statistical Analyses

Before conducting the statistical analyses, the participants’ responses were corrected for input errors (e.g., size in m instead of cm) and checked for plausibility. Implausible or inconsistent answers (e.g., “no experience with glucose measurements” but choosing "daily" for frequency of measurement) were not considered in further analyses. The collected data were analyzed descriptively. Values are expressed as mean values ± standard deviations or as mean values and 95% confidence intervals (95%-CIs). Graphs were prepared using Microsoft Office Excel^®^ 2016 for Windows (Microsoft Corporation, Washington, DC, USA).

### 2.5. Ethical Approval 

The ELSAH project was approved by the local Ethics Committee of the German Sport University Cologne (proposal no. 0342016, date of approval: 04/09/2018). 

## 3. Results

### 3.1. Anthropometric and Sociodemographic Data

A total of 383 subjects participated in the survey. The responses of 316 subjects fulfilled the required 80% completeness, passed the plausibility check and were ultimately included for further analyses. Of the participants, 47.5% were female, 52.2% male and 0.3% diverse. The mean age of the population sample was 38.3 ± 14.5 years (min–max: 18–81 years). Over half of the sample (51.8%) had a higher education, and the monthly net income of 51.3% of the survey participants was between 1000 and 3000 EUR. About 20% earned less than 1000 EUR, and 20% more than 3000 EUR, while 7.7% did not report their monthly net income. 

The mean body mass index (BMI) of the analyzed sample was 24.8 ± 4.5 kg/m^2^ (min–max: 17.3–47.4 kg/m^2^). The participants spent an average of 5.8 ± 4.2 hours (min–max: 0–30 hours) per week on sports or exercise. The majority (75.3%) considered themselves recreational athletes, and 10.6% as elite athletes. The remaining 14.1% declared they did not engage in any physical activities.

Among the subjects, 64 (20.4%) had some form of diabetes mellitus; 70.3% of them had diabetes mellitus type 1. The remaining persons had insulin-dependent (18.8%) or non-insulin-dependent (7.8%) type 2 diabetes mellitus or other forms of diabetes mellitus, such as gestational diabetes (3.1%).

A total of 113 participants (35.8%) reported having experience with lactate measurements. The majority of them (87.9%) performed measurements less than once a month. Of the respondents, 62 (19.9%) stated that they had experience with glucose measurements. In contrast to lactate measurements, glucose measurements were generally performed more frequently among the subjects. Over three-quarters (77.4%) of them performed the measurements daily or several times a week; 14.5% performed them less than once a month. Moreover, 18.5% of the respondents said that they were experienced in using continuous glucose monitoring (CGM) systems.

### 3.2. User Behavior and Acceptance

**Figure 1:**
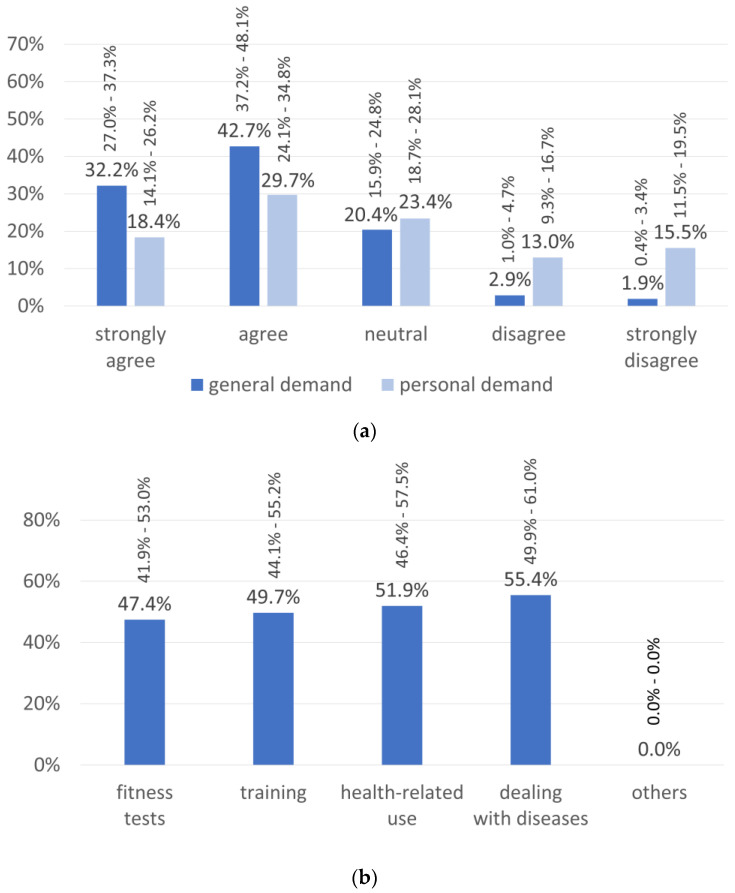
(**a**) Results for the question whether the subjects see a general and/or personal demand for the planned “electronic smart patch system for wireless monitoring of molecular biomarkers for health care and well-being” (ELSAH)-patch system. (**b**) Possible fields of application. Values are expressed as percentages with 95%-CIs.

The participants stated a high general demand for a biochemical sensor system for molecular biomarkers such as the ELSAH-patch, while personal demand was also present but was slightly lower (Figure 1a). According to the participants (multiple answers possible), the ELSAH-patch could be used in fitness tests, for training control, for health-related purposes (e.g., early warning for increased blood glucose levels) and for dealing with diseases (e.g., diabetes mellitus) (Figure 1b).

Respondents who worked as doctors, sports scientists or trainers either “strongly agreed” (31.8%; 95%-CI: 23.7–39.8%) or “agreed” (38.0%; 95%-CI: 29.6–46.4%) that they would also apply the ELSAH-patch to others. A small percentage (0.8%; 95%-CI: 0.0–2.3%) “did not agree”, and 4.7% (95%-CI: 1.0–8.3%) “strongly disagreed” with this statement. The remaining 24.8% (95%-CI: 17.4–32.3%) were “neutral”.

Independently of the price and field of application, 43.8% (95%-CI: 38.3–49.4%) of the participants would use the ELSAH-patch on an occasional (monthly) basis for lactate measurements. It would be used regularly (weekly) by 23.1% (95%-CI: 18.3–27.8%), and 11.0% (95%-CI: 7.5–14.5%) would even use it daily. By contrast, 14.0% (95-CI: 10.1–17.8%) claimed that they would only use it rarely (yearly) or saw absolutely no use in it (8.1%; 95%-CI: 5.1–11.2%). For glucose measurements, 23.9% (95%-CI: 19.2–28.7%) would use the ELSAH-patch permanently (daily), 21.4% (95%-CI: 16.8–25.9%) would use it regularly (weekly) and 32.0% (95%-CI: 26.8–37.2%) occasionally (monthly), while 14.2% (95%-CI: 10.3–18.1%) would rarely use the system (yearly) or not at all (8.4%; 95%-CI: 5.3–11.5%).

Among the subjects, 61.1% (95%-CI: 54.9–67.4%) stated that the perceived pain during blood sampling for analysis using conventional systems did not prevent them from voluntarily performing glucose or lactate measurements. By contrast, the perceived pain did prevent 38.9% (95%-CI: 32.6–45.1%) of the participants from performing measurements, to a low extent (17.9%; 95%-CI: 13.0–22.9%), to some extent (10.7%; 95%-CI: 6.7–14.6%), to a great (8.5%; 95%-CI: 5.0–12.1%) or even to a very great extent (1.7%; 95%-CI: 0.0–3.4%).

The majority of the participants “strongly agreed” (30.4%; 95%-CI: 25.3–35.6%) or “agreed” (34.6%; 95%-CI: 29.3–39.9%) that sustainability (recycling, resource-saving production, etc.) would be a decisive criterion in their decision to buy the ELSAH-patch; 22.3% (95%-CI: 17.7–27.0%) were “neutral” about this question, while 8.7% (95%-CI: 5.6–11.9%) “disagreed” or even “strongly disagreed” (3.9%; 1.7–6.0%) with this statement.

### 3.3. Technical Aspects and Design

**Figure 2:**
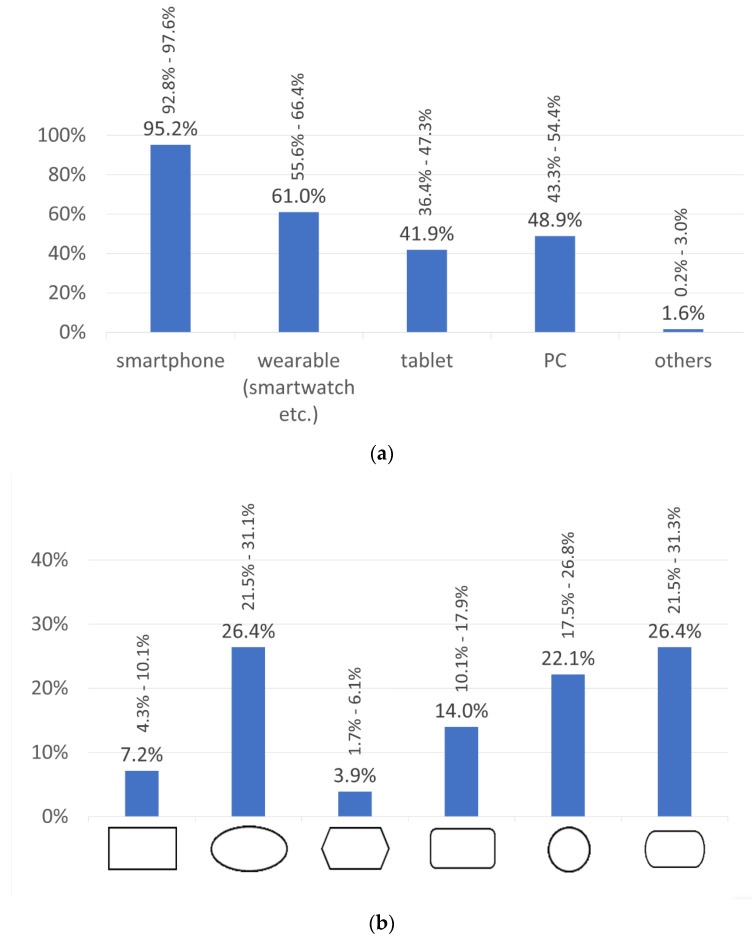
(**a**) Preferred terminal device for use of the ELSAH-patch. (**b**) Preferred shape of the ELSAH-patch. Values are expressed as percentages with 95%-CIs.

Most participants would like to use the ELSAH-patch system in combination with their own mobile phone. The use of a PC or tablet was less popular by comparison, but the option to monitor the measurement values on another wearable (smartwatch, fitness tracker, etc.) was preferred by more than every second person (Figure 2a). Multiple answers were possible for this question.

The respondents would prefer to wear the ELSAH-patch on their upper arm (59.2%; 95%-CI: 53.8–64.7%), where the patch should either be transparent (32.6%; 95%-CI: 27.4–37.8%) or match skin tone (29.1%; 95%-CI: 24.1–34.1%). The most suitable shapes according to the participants are round shapes such as oval, rectangular with rounded ends or circular (Figure 2b).

The ELSAH-patch should be ready to use immediately (32.3%; 95%-CI: 27.1–37.4%) or at least within 30 minutes (45.0%; 95%-CI: 39.5–50.6%). During its operation, 22.6% (95%-CI: 18.0–27.2%) would prefer continuous monitoring (every minute), while 25.5% (95%-CI: 20.7–30.3%) would be satisfied with a 5-minute measuring interval. For 37.3% (95%-CI: 31.9–42.6%) of the participants, an interval of at least 15 minutes would also be sufficient. The length of the interval was “not relevant” for 14.6% of the participants (95%-CI: 10.7–18.6%).

Without information about the planned operational lifetime of the ELSAH-patch, 29.1% (95%-CI: 24.0–34.1%) of the participants preferred a lifetime of up to 12 hours. For 27.5% (95%-CI: 22.5–32.4%), a lifetime of 24 hours would be sufficient. A lifetime of several days was favored by 25.6% (95%-CI: 20.7–30.4%), and 17.9% (95%-CI: 13.6–22.1%) stated that the ELSAH-patch should be operational for more than 1 week. Asked whether a 24-hour lifetime would be sufficient for their specific needs, 55.8% (95%-CI: 50.3–61.3%) agreed. For 73 of the participants (23.4%; 95%-CI: 18.7–28.1%), this life span would be insufficient. The remaining participants (20.8%; 95%-CI: 16.3–25.3%) neither agreed nor disagreed.

Asked which other biomarkers would be of interest to the respondents and which should be measured by the ELSAH-patch (multiple answers possible), inflammatory markers (74.7%; 95%-CI: 69.8–79.7%), markers for myocardial infarction (56.9%; 95%-CI: 51.3–62.5%), hormones (51.2%; 95%-CI: 45.5–56.9%) and oncological factors/cancer markers (46.1%; 95%-CI: 40.5–51.8%) were selected from the listed markers. For the option to add unlisted markers, atherosclerotic markers, ketones and biomarkers for hydration were mentioned.

### 3.4. Software and Data Security

In response to the question how the collected measurement values should be presented in the software (multiple answers possible), 75.7% (95%-CI: 70.9–80.5%) of participants preferred a comparison with previous measurement values or an assessment of whether the values are/are not critical to their health (69.3%, 95%-CI: 64.1–74.4%). A comparative visualization with data from a reference group was supported by 54.4% (95%-CI: 48.8–59.9%), and 50.5% (95%-CI: 44.9–56.1%) would like to have the possibility of inspecting the raw data.

**Figure 3:**
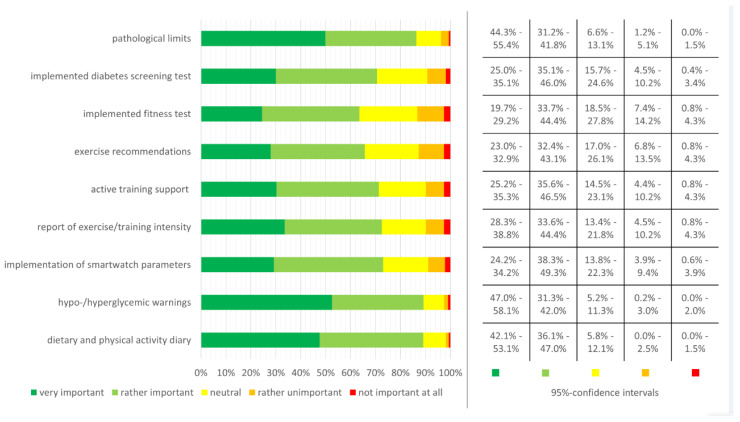
Importance of potential functions and features included in the ELSAH-patch software. Corresponding 95%-CIs are shown on the right side of the figure.

Participants were asked to rate potential functions and features that could be implemented in the ELSAH-patch software (Figure 3). Information on pathological limits and warnings for hypo-/hyperglycemic conditions as well as a diary function for diet and physical activity were considered the most important functions.

Data security was also a very important factor for 69.8% (95%-CI: 64.8–74.9%) of the respondents and at least an important criterion for 17.8% (95%-CI: 13.6–22.0%). Only a minority of 2.5% (95%-CI: 0.8–4.3%) did not consider data security to be important. Accordingly, 52.1% (95%-CI: 46.5–57.6%) “strongly agreed” and 31.7% (95%-CI: 26.6–36.9%) “rather agreed” that a high level of data security (e.g., end-to-end encryption, encrypted data storage, etc.) would influence their decision to buy the ELSAH-patch system. On the other hand, 86.1% (95%-CI: 82.3–89.9%) would also agree to allow specific persons (e.g., doctors, family members and/or trainers) to access their data with their explicit consent.

### 3.5. Pricing

The participants were asked how much money they would be willing to spend per month on average for the complete ELSAH-patch system. Slightly over half of the respondents (52.5%; 95%-CI: 47.0–58.1%) would pay between 20 and 100 EUR per month, whereas 35.0% (95%-CI: 29.8–40.3%) would spend less than 20 EUR. Some respondents (6.4%; 95%-CI: 3.7–9.1%) would even be willing to spend 100–200 EUR, while none of the participants would spend more than 200 EUR. Other participants (6.1%; 95%-CI: 3.4–8.7%) would not buy the ELSAH-patch system at all.

According to the survey results, the maximum price for a single ELSAH-patch, if the lifetime was 24 hours and the software was free of charge, should not exceed 10 EUR for 76.5% of the respondents (95%-CI: 71.8–81.2%); 12.7% (95%-CI: 9.0–16.4%) would pay a maximum price of between 10 and 15 EUR, and 4.4% (95%-CI: 2.2–6.7%) would even pay over 15 EUR. In this context, 6.3% of the participants (95%-CI: 3.7–9.0%) answered that they would not buy the ELSAH-patch system under the mentioned conditions.

If a single ELSAH-patch would cost 15 EUR, the corresponding software/mobile app with all the previously defined specifications should not cost more than 5 EUR for 28.6% (95%-CI: 23.6–33.6%) of the participants. Around one-third (30.2%; 95%-CI: 25.1–35.2%) would be willing to spend between 5 and 15 EUR for the software. Another 30.8% (95%-CI: 25.7–35.9%) would even pay more than 15 EUR for the software, while 10.5% (95%-CI: 7.1–13.9%) would not pay for the software/mobile app under these conditions. 

In response to the open-ended question what other factors would be important in relation to the ELSAH-patch, 39 participants suggested considering aspects such as:

- ELSAH-patches should be waterproof/should not detach when sweating (even during high-intensity sports);
- ELSAH-patches should be easily available (short delivery time);
- Cost absorption by health insurance would be desirable in case of diseases (use as a medical device);
- There should be technical support/a hotline;
- The ELSAH-patch system should be easy to use (patch and software);
- Export options for data should be provided/data transfer to other platforms/apps should be possible.

## 4. Discussion

The survey results show high user acceptance for the ELSAH-patch system. The participants would prefer to use it for personal health care, but also identified possible applications within the field of physical activity/training. In general, the results show good congruence with the intended specifications of the ELSAH-patch on factors such as accuracy and data security, which should be implemented accordingly. The results of this study also provide important information for developers on aspects such as operational lifetime or water/sweat resistance, which should be taken into account.

The high demand for such a system is in line with the immense interest in wearable devices for personal health care, fitness and well-being [19]. The participants would use the ELSAH-patch in different areas of application. Although the results do not indicate a clear focus, the tendency seems to be towards more frequent use for health-related aspects. Wearables that are already available on the market to measure physical activity or the heart rate could contribute to the user’s health [20,21,22], but biochemical sensors that continuously monitor biomarkers offer a more detailed perspective of an individual’s physiological state [1]. Therefore, the ELSAH-patch system has considerable potential in the personal health care segment.

Medical/well-being aspects, e.g., guidance on pathological limits or warnings for hypo-/hyperglycemic conditions, were more important for the respondents than support functions during physical activity/training. Therefore, high standards of accuracy and reliability are important. Measurement accuracy is essential, especially for low or rapidly changing glucose concentrations, and is still a major challenge for existing sensors [23]. The first version of the ELSAH-patch should measure lactate and glucose concentrations with a precision of 0.1 mmol/L and an accuracy with a mean absolute relative difference (MARD) (compared to reference measurements in the blood) of not more than 15%. Established systems for continuous glucose monitoring lie within a similar range or slightly below [24,25]. Other minimally or non-invasive systems, partly still under development, are occasionally unable to reach a target of 15% MARD [26]. However, this shows how important it is for the ELSAH project team to achieve the ambitious target and that improvements in the accuracy of the system could be decisive for achieving a competitive advantage in the market.

The operating lifetime of the ELSAH-patch was planned to be 24 h. This would be sufficient for 55.8% of the respondents. However, especially for people with diabetes mellitus, continuous glucose monitoring over a long period of time is, of course, important for managing the disease. Therefore, a relatively high number of people with diabetes were among the 23.4% (95%-CI: 18.7–28.1%) of participants for whom a 24-hour lifetime would not be sufficient. The balancing act regarding users’ expectations and technical feasibility is a tremendous challenge for the ELSAH system developers. In a comparable survey on the general expectations for wearable and implantable sensors, 37% of the respondents stated that they expected a battery life of more than 6 months [17]. Such expectations are hardly compatible with other expectations, e.g., that the sensor should be discreet and compact. However, the results show that the intended lifetime of the ELSAH-patch is sufficient for most of the applications. Nevertheless, developers should pay attention to an efficient and energy-saving operation of the ELSAH-patch to optimize and possibly extend the battery lifetime. In addition, technical aspects such as multi-source energy harvesting systems (e.g., biofuel cells), which could lead to a longer energy supply, should be taken into account [9,27]. A longer operating lifetime for the ELSAH-patch could open up new potential fields of application.

Daily life conditions are a further challenge for the ELSAH-patch. Preventing early sensor detachment is probably one of the most difficult tasks for developers. Accordingly, the participants mentioned this aspect in the open-ended question at the end of the questionnaire: the sensor should be waterproof and/or should not detach under conditions of sweating. Demands for the adhesion of the ELSAH-patch are high, especially when having a shower or swimming or during intensive physical activity (heavy sweating). The function of the ELSAH-patch system should not be restricted by these daily life conditions. In this context, it is important that the chosen adhesive ensures reliable operation without early detachment while avoiding skin irritations or allergic reactions already observed for other patch-based glucose-monitoring systems [28].

Another very important issue for the ELSAH project will be ensuring data security. The potential end-users have high demands as regards the protection of their personal data. Since health-related information about glucose and lactate levels collected by the ELSAH-patch is highly sensitive, the misuse of data must be prevented. Self-perceived privacy control and trust have been identified as key factors for the intention to use health-related wearables in a former survey examining the decisions to use personal health record systems by young U.S. individuals [29]. This is in line with the results showing that a high level of data security would influence the respondents’ decision to extensively use the ELSAH-patch system. Nonetheless, participants would be willing to share their data with others, e.g., with their doctors. Therefore, it is important for users to be able to securely and easily manage external access and that their data are protected from unauthorized access or unwanted disclosure. Many mobile health applications fail in this regard, whereby a lack of encryption, insecure data transmission or inadequate programming practices are some of the major security concerns [30,31]. The ELSAH project ensures high security standards in accordance with the General Data Protection Regulation (GDPR) of the European Union. 

For a complete profiling of a person’s well-being, a variety of parameters must be detected simultaneously. As the concept of the ELSAH-patch can easily be extended to the multiplexed detection of additional biomarkers, the participants were asked which other biomarkers would be of interest. Although the ELSAH project will initially focus on continuous measurement of glucose and lactate, it was important to obtain strategic information for the future market and developments. 

The results of the present survey/the end-users´ demands and expectations should be taken into account as far as possible in the development of the ELSAH-patch system. Users´ demands, for example, in terms of security, can be further addressed by encrypted end-to-end data transmission from the ELSAH-patch to the terminal device and encrypted data storage. User authentication and "privacy by design" and "privacy by default" principles should guarantee a high level of privacy and security. Furthermore, the users´ expectations in terms of tracking several other molecules/biomarkers could also be fulfilled. The concept of the ELSAH-patch allows adding additional electrodes with specific enzymes for the quantification of further biomarkers. After evaluating the possibilities of the production companies, the respondents´ wishes, for example, with regard to the shape and color of the patches, should also be considered.

### Limitations

The present study sample is not representative in relation to Germany’s total population. In a representative survey on health and physical activity in Germany with 2885 participants, at least half of the respondents stated that they were not physically active in their leisure time [32]. 

In this study, non-active persons (14.1%; 95%-CI: 10.2–18.0%) represented only a small proportion of the sample. Although the analyzed sample in this study is different from the total population, it can be assumed that the ELSAH-patch target group could have comparable characteristics, as the ELSAH project primarily targets personal health care, fitness and well-being, and the majority of the potential end-users are expected to be very health-conscious.

## 5. Conclusions

The intended specifications of the ELSAH-patch correspond well with the results of the survey on the expectations and requirements of possible end-users. The findings confirm the high relevance of a minimally invasive smart patch system for the wireless monitoring of molecular biomarkers and indicate high user acceptance for its use in health care, fitness and well-being applications. Over half of the respondents would pay between 20 and 100 EUR per month for such a system. The study’s results emphasize the importance of the ambitious standards for accuracy and data security and their consequent implementation. In addition, important information on operational lifetime and water/sweat resistance was obtained, which should be taken into account in the development process. 

## Supplementary Materials

The following data files are available online at https://www.mdpi.com/1424-8220/20/20/5761/s1: ELSAH-questionnaire.
