# Supplementary material for: Minimally Invasive Electrochemical Patch-Based Sensor System for Monitoring Glucose and Lactate in the Human Body—A Survey-Based Analysis of the End-User’s Perspective
Source: Sensors (Basel). 2020 Oct 11;20(20):5761. doi: 10.3390/s20205761 (PMC7601713; doi:10.3390/s20205761)
Supplement: Supplementary file 1 [file sensors-20-05761-s001.pdf]

1. Anthropometric and sociodemographic data

Age: _______ years Size: _______ cm	Weight: _______ kg
Gender: □ female □ male □ diverse	Profession: _______________________
What is your highest level of education?	□ school completed, without graduation<br>□ secondary school certificate<br>□ secondary school leaving certificate<br>□ A-levels (or university entrance qualification)<br>□ university degree (Bachelor, Master)<br>□ academic title (Dr., PhD, Prof. Dr.)
What is your monthly net income?	□ less than 1000 EUR<br>□ 1000 EUR - 2000 EUR<br>□ 2000 EUR - 3000 EUR<br>□ 3000 EUR - 4000 EUR<br>□ more than 4000 EUR<br>□ no response
How would you rate yourself?	□ elite athlete<br>□ recreational athlete<br>□ non-active person
Do you work as a trainer/sports coach?	□ yes □ no
How many hours a week do you perform physical exercise?	________ hours / week
How would you rate your diet?	□ healthy diet<br>□ partly healthy, party unhealthy<br>□ unhealthy diet
Do you smoke?	□ yes □ no
Have you been diagnosed with diabetes mellitus?	□ no □ type 2 insulin-dependent<br>□ type 1 □ type 2 non-insulin-dependent<br>□ other type (e.g. gestational diabetes)
Do you have any experience with lactate measurements?	□ yes □ no
Do you have any experience with lactate measurements on other people?	□ yes □ no
**If you answered yes to either of the last two questions (otherwise, please skip this question):**<br>How often do you perform lactate measurements?	□ daily<br>□ weekly, but not every day<br>□ several times a month, but not every week<br>□ once a month<br>□ less than once a month

Do you use blood glucose test strips to monitor/control your blood glucose?	□ yes □ no
Do you use blood glucose test strips to monitor/control other people's blood glucose?	□ yes □ no
**If you answered yes to either of the last two questions (otherwise, please skip this question):**<br>How often do you measure blood glucose?	□ daily<br>□ weekly, but not every day<br>□ several times a month, but not every week<br>□ once a month<br>□ less than once a month
Do you use continuous blood glucose measurement systems (e.g. Freestyle Libre2, Dexcom G6, Enlite, etc.)? NOT blood glucose test strips!	□ yes □ no

Brief description of the project - Please read the following information carefully!

Within the scope of the EU-funded project "ELSAH", a patch for the minimally-invasive measurement of biomolecules in the human body (especially glucose and lactate) is being developed. The use of this patch is painless.

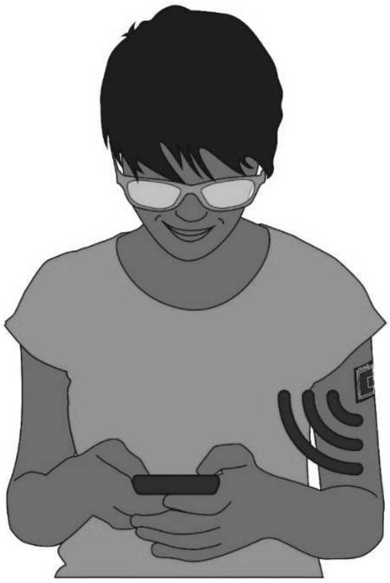

Figure: ELSAH-patch for the measurement of biomolecules

Monitoring your glucose concentration using the ELSAH-patch could help you detect diabetes mellitus at an early stage, or in case of existing diabetes mellitus, to regulate your blood sugar concentration. Measuring lactate using the patch could help you determine your fitness level and/or identify your optimal intensity zone for your physical exercises. The patch can communicate wirelessly with your mobile phone (in combination with a software (app) on your mobile phone). No complex calibration process is required. Glucose and lactate values are continuously recorded at regular intervals. The patch can be disposed together with household waste. There are no risks/hazards for the user during its application.

2. User behavior/User acceptance

	**strongly agree**	**agree**	**neutral**	**disagree**	**strongly disagree**
I have a personal need for such a product.	□	□	□	□	□
There is a general need for such a product.	□	□	□	□	□
If you belong to one of the following professions: sports scientist, trainer, doctor:<br>I would apply the ELSAH-patch system to others.	□	□	□	□	□
Sustainability (recycling, resource-saving production, etc.) would be a decisive criterion for me to buy the ELSAH-patch system.	□	□	□	□	□
In which situations would you apply the ELSAH-patch system yourself?<br>Multiple answers possible!	□ fitness tests<br>□ during training (e.g. for training control)<br>□ health-related use<br>(e.g. early warning of diabetes mellitus)<br>□ in dealing with diseases<br>(e.g. diabetes mellitus)<br>□ others: ______________________				
How often would you use the ELSAH-patch system for lactate measurements, regardless of its price?	constantly (daily)<br>□	regularly (weekly)<br>□	occasionally (monthly)<br>□	rarely (yearly)<br>□	never<br>□
How often would you use the ELSAH-patch system for glucose measurements, regardless of its price?	constantly (daily)<br>□	regularly (weekly)<br>□	occasionally (monthly)<br>□	rarely (yearly)<br>□	never<br>□
Please answer only if you have been regularly measuring your lactate and/or glucose values:<br>How would you rate the level of pain during measurements?	extremely painful<br>□	very painful<br>□	painful<br>□	slightly painful<br>□	not at all painful<br>□
To what extent did the pain of using conventional systems prevent you from voluntarily measuring your lactate and/or glucose values?	very great extent<br>□	great extent<br>□	some extent<br>□	little extent<br>□	not at all<br>□
Have you ever had any allergic reaction when wearing patches?	□ yes □ no				
**If yes**, what brand / type triggered the allergic reaction?	□ following product: ____________________<br>□ can't remember				

3. Technical aspects and design

The ELSAH patch should be able to transfer the data to a ...<br>Multiple answers possible!	□ smartphone<br>□ tablet<br>□ PC<br>□ wearable (smartwatch, fitness tracker, etc.)<br>□ other: ____________________________
Where on the body would you say is the best place to apply the patch?<br>Please choose **one** option!	□ forearm<br>□ upper arm<br>□ abdomen<br>□ back<br>□ not relevant<br>□ upper leg<br>□ lower leg<br>□ neck<br>□ buttocks
For your application: how quickly does the system have to be ready-to-use (from the time the patch is applied to the first valid reading)?	□ immediately<br>□ within 30 minutes<br>□ within 1 hour<br>□ within 2 hours<br>□ not relevant
For your application: at what intervals should the ELSAH-patch system provide data?	□ every minute<br>□ every 5 minutes<br>□ every 15 minutes<br>□ every 30 minutes<br>□ not relevant
For your application: for how long should the ELSAH-patch system be able to operate?	□ less than 12 hours<br>□ 12 hours<br>□ 24 hours<br>□ several days<br>□ more than one week
A 24-hour patch lifetime would be sufficient for my application.	strongly agree □ | agree □ | neutral □ | disagree □ | strongly disagree □
What patch shape would you prefer?<br>Please choose **one** option!	□ □ □ □ □ □ 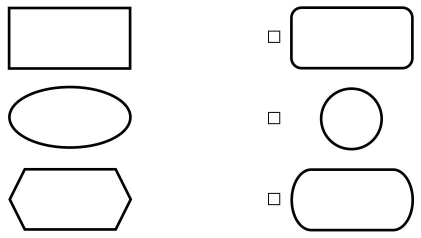<br>□ not relevant
Which color would you prefer?<br>Please choose **one** option!	□ transparent<br>□ white<br>□ skin tone<br>□ colored<br>□ not relevant
What other biomarkers would you be interested in / should ELSAH be capable of measuring in the future?<br>Multiple answers possible!	□ inflammatory factors<br>□ hormones<br>□ oncological factors/cancer markers<br>□ marker for myocardial infarction<br>□ others: ______________________

4. Software and data security

How should the measured values be displayed?<br>Multiple answers possible!	□ raw data (glucose and/or lactate values)<br>□ analyzed data (notification about possible pathological alterations)<br>□ compared with reference values (trends in relation to pre-test values, reference group values)<br>□ compared with own previous measurements (trends)<br>□ others: ________________________

**How important is it for you…**	**very important**	**rather important**	**neutral**	**rather unimportant**	**not important at all**
… that the software provides information about thresholds above which the values are considered a health risk?	□	□	□	□	□
… that the software offers a diabetes mellitus screening test that can be performed using the ELSAH-patch system?	□	□	□	□	□
… that the software offers a fitness test that can be performed using the ELSAH-patch system?	□	□	□	□	□
… that the software provides recommendations for optimal training intensity ranges (based on the fitness test)?	□	□	□	□	□
… that the ELSAH-patch system informs you whether you are within your optimal training range during your workout?	□	□	□	□	□
… that the ELSAH-patch system provides information about the intensity of your overall workout once you have finished it?	□	□	□	□	□
… that the software/app can display/process other parameters (e.g. heart rate, step count, etc.) of other health-related wearable devices?	□	□	□	□	□
… that the software / app informs/warns you about/of (imminent) critical blood values (hypoglycemia or hyperglycemia)?	□	□	□	□	□
… that the software/app contains a history/diary function for the measured values?	□	□	□	□	□
… that the system ensures a high level of data security/privacy?	□	□	□	□	□

	strongly agree	agree	neutral	disagree	strongly disagree
A high level of data security (end-to-end encryption, encrypted data storage, etc.) would influence my decision to buy the ELSAH-patch system.	□	□	□	□	□
I would allow certain persons (e.g. family members and/or my doctor and/or trainer) to access my data (only with my explicit consent).	□	□	□	□	□

5. Pricing

How much money would you be willing to spend on the ELSAH-patch system per month on average (with the ability to measure both lactate and glucose)?<br>Total costs, including patches and software (based on all of the requirements you specified previously).	□ less than 20 EUR<br>□ 20-100 EUR<br>□ 101-200 EUR<br>□ over 200 EUR<br>□ I would not buy the ELSAH-patch system
What is the maximum amount you would pay for a single application of an ELSAH-patch if its lifetime was 24 hours and the software (based on all of the requirements you specified previously) was free of charge?	□ less than 5 EUR<br>□ 5-10 EUR<br>□ 11-15 EUR<br>□ over 15 EUR<br>□ I would not buy the ELSAH-patches
If the software was not free of charge, what would be the maximum one-time fee you would pay for the software/app (based on all of the requirements you specified previously), if the patches had a final price of 15 EUR per patch?	□ less than 5 EUR<br>□ 5-15 EUR<br>□ 16-25 EUR<br>□ over 25 EUR<br>□ I would not buy the ELSAH-patch software

What other factors associated with the ELSAH-patch system are of relevance to you? Additional comments:

Thank you very much for your participation!
